# Radiation-induced alternative transcripts as detected in total and polysome-bound mRNA

**DOI:** 10.18632/oncotarget.21672

**Published:** 2017-10-09

**Authors:** Amy Wahba, Michael C. Ryan, Uma T. Shankavaram, Kevin Camphausen, Philip J. Tofilon

**Affiliations:** ^1^ Radiation Oncology Branch, National Cancer Institute, Bethesda, MD 20892, USA; ^2^ In Silico Solutions, Falls Church, VA 22043, USA

**Keywords:** radiation, polysomes, alternative splicing, gene expression

## Abstract

Alternative splicing is a critical event in the posttranscriptional regulation of gene expression. To investigate whether this process influences radiation-induced gene expression we defined the effects of ionizing radiation on the generation of alternative transcripts in total cellular mRNA (the transcriptome) and polysome-bound mRNA (the translatome) of the human glioblastoma stem-like cell line NSC11. For these studies, RNA-Seq profiles from control and irradiated cells were compared using the program SpliceSeq to identify transcripts and splice variations induced by radiation. As compared to the transcriptome (total RNA) of untreated cells, the radiation-induced transcriptome contained 92 splice events suggesting that radiation induced alternative splicing. As compared to the translatome (polysome-bound RNA) of untreated cells, the radiation-induced translatome contained 280 splice events of which only 24 were overlapping with the radiation-induced transcriptome. These results suggest that radiation not only modifies alternative splicing of precursor mRNA, but also results in the selective association of existing mRNA isoforms with polysomes. Comparison of radiation-induced alternative transcripts to radiation-induced gene expression in total RNA revealed little overlap (about 3%). In contrast, in the radiation-induced translatome, about 38% of the induced alternative transcripts corresponded to genes whose expression level was affected in the translatome. This study suggests that whereas radiation induces alternate splicing, the alternative transcripts present at the time of irradiation may play a role in the radiation-induced translational control of gene expression and thus cellular radioresponse.

## INTRODUCTION

The development of molecularly targeted approaches for enhancing the efficacy of radiotherapy, a primary treatment modality for most solid tumors, requires a comprehensive understanding of the processes comprising cellular radioresponse. Along these lines, fundamental determinants of radioresponse, such as DNA repair and cell cycle checkpoint activation, have been best described in terms of the post-translational modification of existing proteins. Modifications in gene expression, however, are also a consequence of irradiation suggesting a role for de novo protein synthesis in determining cellular radioresponse. Towards understanding the biological significance of radiation-induced gene expression, studies initially focused on defining the changes in the transcriptome (total cellular mRNA) in irradiated cells [1-6]. However, interpreting the significance of such transcriptome analyses is complicated by the lack of correlation between the radiation-induced changes in mRNA and their corresponding proteins [7]. To address the discrepancy between the radiation-induced transcriptome and proteome, polysome-bound RNA, which identifies genes undergoing translation and defines the translatome, was subjected to microarray analysis and compared to the traditional microarray approach using total cellular mRNA [8, 9]. The results of these studies showed that the number of genes in the radiation-induced translatome was substantially greater than those in the radiation-induced transcriptome and that there was little to no overlap between the two expression profiles. Moreover, in contrast to the changes in the transcriptome after irradiation, there was a strong correlation between genes appearing in the radiation induced translatome and changes in the levels of their corresponding proteins. Thus, data indicate that radiation modifies gene expression primarily through translational control.

One of the critical events in the post-transcriptional regulation of gene expression is the splicing of introns from precursor mRNA (pre-mRNA) [10]. Splicing can be the same in each pre-mRNA from a given gene or can proceed through alternative splicing events resulting in the generation of mRNA isoforms (alternative transcripts) from the same genomic sequence. Most human genes undergo alternative splicing (AS), which is recognized as a major source of protein diversity [11-13]. A well-established mechanistic consequence of AS is the formation of transcripts with increased susceptibility to nonsense mediated decay, which ultimately reduces gene expression [12-14]. However, in HEK293T cells and in mouse embryonic stem cells the association of mRNAs with polysomes has been reported to occur in an isoform specific manner [15, 16], suggesting a role for AS in the translational control of gene expression.

Radiation has been shown to induce AS in the evaluation of individual genes [17-19] and at the whole transcriptome level using exon arrays [20-22]. Whether AS plays a role in radiation-induced translational control of gene expression has not yet been reported. In the study described here we used RNA-Seq and the bioinformatics program SpliceSeq to identify alternative transcripts in the transcriptome and translatome after irradiation of a glioblastoma stem-like cell line. Data presented here show that radiation induced substantially more AS events in the polysome fraction (translatome) as compared to total cellular RNA (transcriptome) with only a minor overlap between the 2 fractions. Moreover, many of the genes corresponding to splice events unique to the radiation-induced translatome were associated with DNA repair processes. These results suggest that radiation not only induces AS, but that there is also a preferential recruitment of specific mRNA isoforms to polysomes after irradiation, suggesting a role for AS in the radiation-induced translational control of gene expression.

## RESULTS

We recently described the radiation-induced transcriptomes (total cellular mRNA) and translatomes (polysome-bound mRNA) for the NSC11 glioblastoma stem-like cell (GSC) line along with 2 additional GSC lines using microarray analysis [9]. In that study, radiation-induced changes in polysome-bound mRNA correlated with changes in corresponding proteins and associated cell processes supporting the concept that radiation-induced translational control of gene expression is a fundamental component of radioresponse. To build on these data and as an initial investigation into the potential role of alternative splicing (AS) in the radiation-induced translational control of gene expression, we used RNA-Seq and SpliceSeq analyses on untreated and irradiated NSC11 cells. Rather than using a single parameter such as RPKM (gene reads per kilobase of transcript per million aligned reads), identification of significant AS events using SpliceSeq was based on the combination of 5 criteria (as listed in Methods), which included the necessity of being detected in 5 of the 6 biological replicates.

### Alternative splice events specific to polysome-bound RNA in untreated cells

**Figure 1:**
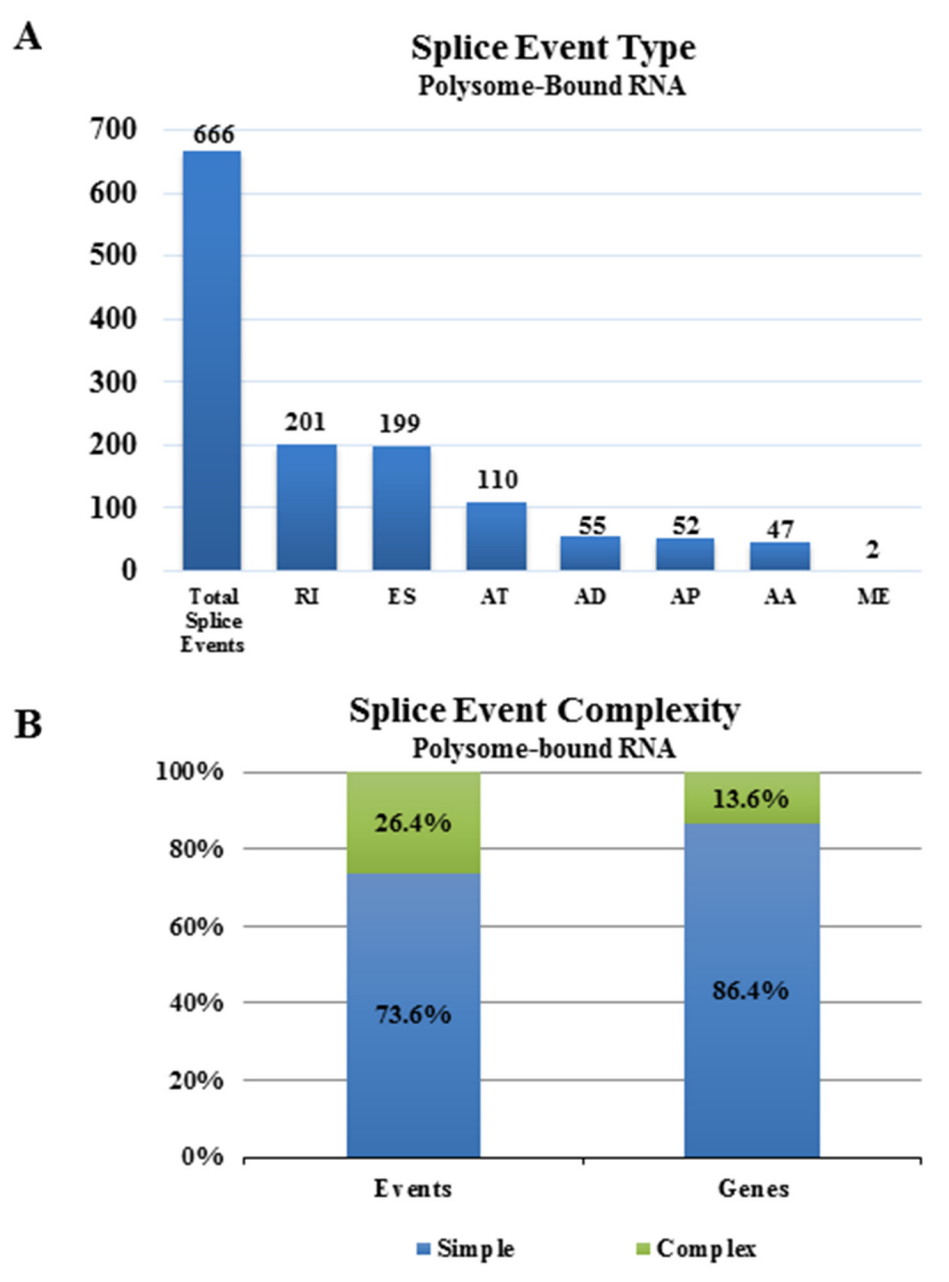
Alternative splice events in the translatome as compared to the transcriptome **(A)** Numbers of significant alternative splice events in the translatome of NSC11 as compared to the transcriptome according to category [alternate acceptor (AA), alternate donor (AD), alternate promoter (AP), alternate terminator (AT), exon skip (ES), mutually exclusive exons (ME), retained intron (RI)]. **(B)** Complexity of splice events in polysome-bound RNA as compared to total RNA. Simple (blue) refers to 1 event and complex (green) refers to more than 1 event.

Initially, we compared the alternative transcript diversity in polysome-bound versus total RNA from untreated NSC11 cells. Specifically, from both polysome-bound RNA (isolated by sucrose gradient fractionation) or total cellular RNA, polyA^+^ selected RNA was subjected to paired-end sequencing with mapping to the human hg19 reference genome (see Supplementary Table 1 for RNA-Seq parameters); we then used the comparison option of SpliceSeq to identify genes with splicing differences between polysome-bound mRNA (the translatome) and total cellular mRNA (the transcriptome). Comparison of mRNA isoforms detected in polysome-bound mRNA to those in total mRNA revealed 666 splicing events significantly enriched in the polysome-bound RNA (Figure 1A) corresponding to 567 genes (Supplementary Table 2). The splice events specific to polysomes divided into 7 types with more than half attributed to mRNA with alternatively retained introns (RI) and mRNA that had alternative exon skip (ES) events and about 20% that had an alternative termination (AT). The splice events specific to polysome-bound mRNA primarily involved one splice event per gene (simple events) with most of the affected genes containing only a single event, suggesting that the single splice event was sufficient to change the polysome association of most of the identified transcripts in untreated samples (Figure 1B). Genes containing more than one splice event per gene (complex) comprised only 13.6% of the genes undergoing alternative splicing. The selective association of alternative transcripts with polysomes suggests that AS influences RNA translation in NSC11 cells, consistent with results obtained from other cell lines [15, 16].

### Radiation-induced alternative transcripts

**Figure 2:**
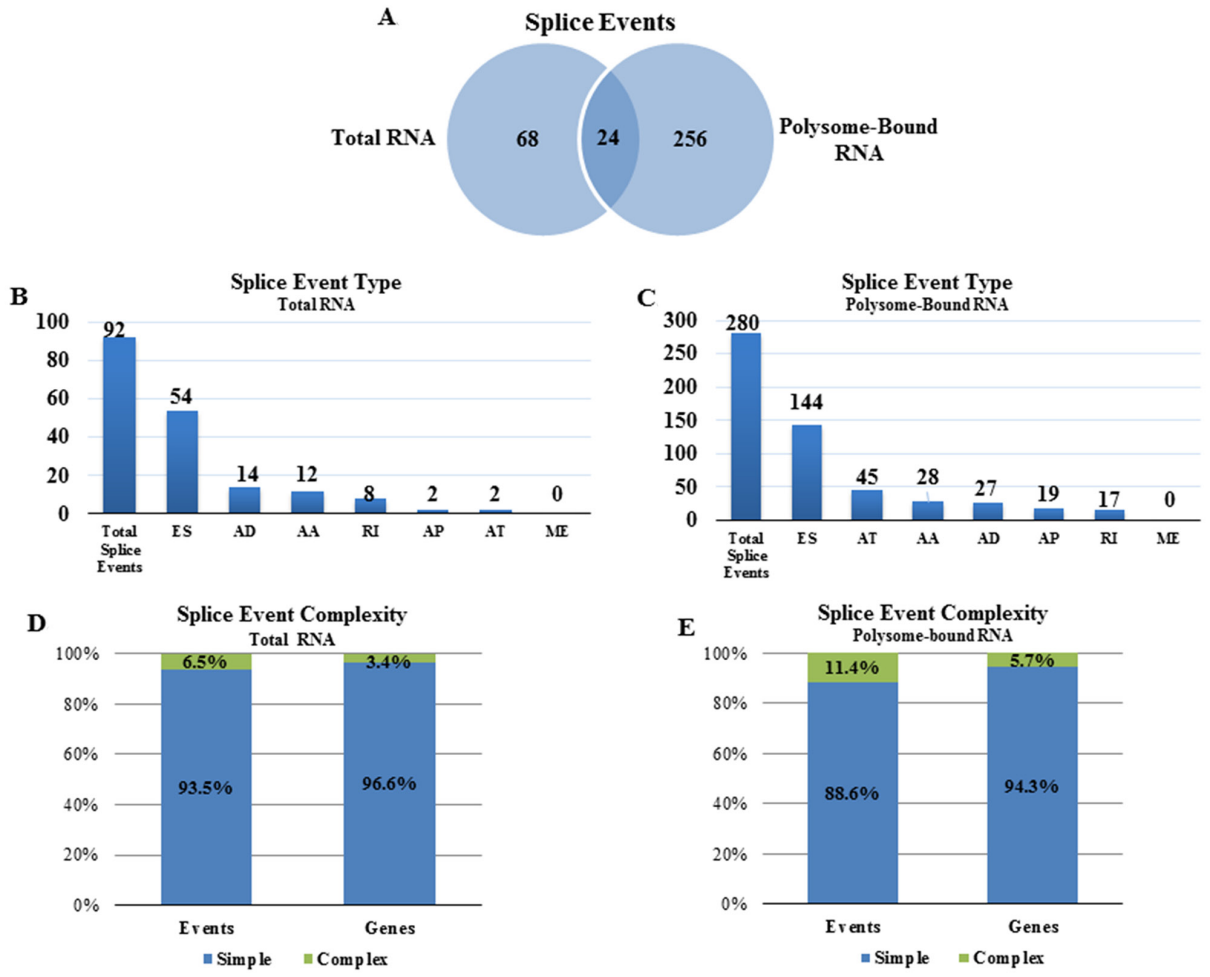
Radiation-induced alternative splice events in the transcriptome and the translatome **(A)** Venn diagram comparing the number of radiation-induced alternative splice events in total versus polysome-bound RNA. Number of radiation-induced splice events in **(B)** total RNA and **(C)** polysome-bound RNA according to category as defined in Figure 1. Complexity of radiation-induced splice events in the **(D)** transcriptome and **(E)** translatome. Simple (blue) refers to 1 event and complex (green) refers to more than 1 event.

To investigate the effects of radiation on AS, total cellular mRNA or polysome-bound mRNA was collected from NSC11 cells 1h after irradiation (2 Gy) or after mock irradiation (control) and subjected to RNA-Seq. An advantage provided by using NSC11 cells is that the majority of changes in the translatome occur at 1h after irradiation [9], which reduces the potential contribution of transcriptional modifications allowing for a more direct investigation of the role of AS in the translational control of gene expression [23]. For each mRNA compartment, the comparison option of SpliceSeq was used to identify genes with splicing differences between control and irradiated cells. Splice events enriched in total cellular mRNA or polysome-bound mRNA after irradiation are shown in Figure 2A. Ninety-two splice events were detected in the radiation-induced transcriptome (total RNA), which, consistent with previous studies using exon arrays [20, 21], suggests that radiation induces AS of pre-mRNA. However, 280 splice events were detected in the radiation-induced translatome (polysome-bound RNA) of which only 24 were overlapping with the radiation-induced transcriptome. Given the discrepancy between radiation-induced splice events in the translatome and transcriptome, it appears that radiation not only modifies AS of pre-mRNA, but also results in the selective association of existing mRNA isoforms with polysomes. The types of radiation-induced splice events appeared to be similar between the translatome and transcriptome with more than half being of the exon skipping (ES) form in each compartment (Figure 2B and 2C). The vast majority of splice events induced by radiation in both the transcriptome and translatome analyses were classified as simple events (one splice event per gene) with most of the affected genes containing only one event (Figure 2D and 2E). These results suggest that a single splicing event is sufficient to affect the radiation-mediated enrichment of an mRNA in the polysome-bound compartment.

### Validation of splice events

**Figure 3:**
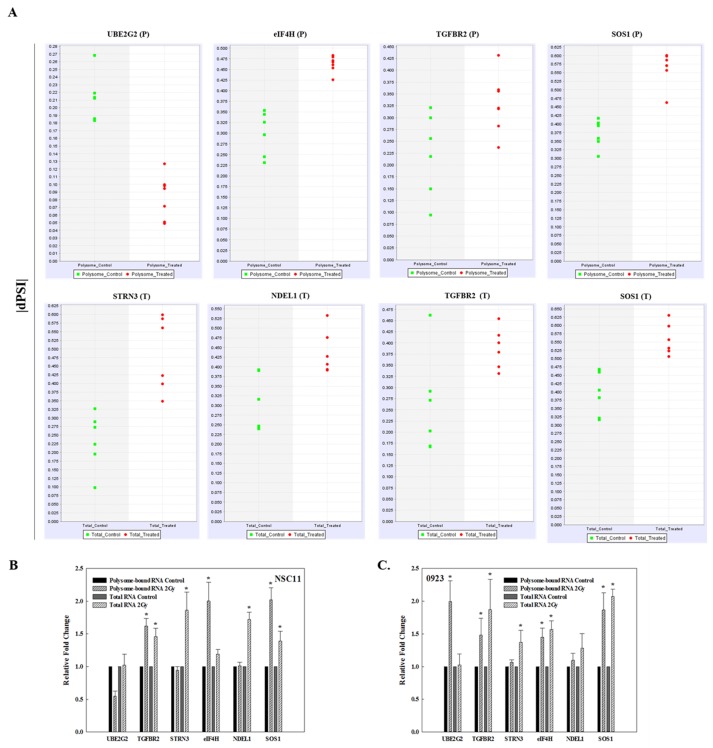
Validation of radiation-induced alternative transcripts in total and polysome-associated RNA **(A)** dPSI (change in percent spliced in) scatter plots for the individual replicates of mock irradiated (control, green) and irradiated (2 Gy, 1h, red) in polysome-associated RNA (P) and total RNA (T). qRT-PCR of polysome-bound RNA and total RNA of the indicated gene normalized to 18S rRNA in **(B)** NSC11 cells and **(C)** 0923 cells. Each value represents the mean ± SEM for 3 independent experiments. ^*^p < 0.05 according to Student’s *t*-test (radiation vs. control).

The presence of the radiation-induced splice events was validated using qRT-PCR. The 6 genes evaluated involved radiation-induced splice events enriched in polysome-associated RNA only (UBE2G2, eIF4H), total cellular RNA only (STRN3, NDEL1), and splice events common to both (TGFBR2, SOS1). These genes were selected for evaluation because they contained among the largest RPKMs for alternative exon skip events, the most common radiation-induced splice event detected in the transcriptome and translatome; were predicted to be modified in only the translatome, only the transcriptome or both and for which there were commercially available primer assays. Primers were selected based on sequence analysis to flank a splice junction of the transcript as predicted by SpliceSeq. This allowed detection of the changes in each transcript predicted to be alternatively spliced after irradiation and thus to reflect changes in the splice event rather than transcript abundance. The dPSI (change in percent spliced in and a measure of splice frequency) for each of these splice events in the individual replicates from each treatment condition show the change in splicing after irradiation as well as a high reproducibility across the 6 replicates (Figure 3A). The qRT-PCR analysis (Figure 3B) indicates the same qualitative changes induced by radiation as detected by RNA-Seq/SpliceSeq with the spliced exon of UBE2G2 reduced in polysome-bound RNA after irradiation compared to a constant value in total RNA after irradiation. The spliced exon in eIF4H increased in the polysome fraction after irradiation and in NDEL1 increased in the total RNA fraction after irradiation, both consistent with the RNA-Seq/SpliceSeq data. As predicted, the spliced exon in STRN3 increased in the total RNA fraction in response to radiation and in TGFBR2 and SOS1, the spliced exon increased in both polysome-bound RNA and total RNA. Thus, the qRT-PCR results validate the RNA-Seq/SpliceSeq analyses of radiation-induced alternate transcripts in total and polysome-bound mRNA. To determine whether the splice events induced by radiation were specific to NSC11 cells, qRT-PCR analysis was performed using the GSC line, 0923. As shown in Figure 3C, for 3 of the 6 genes evaluated (TGFBR2, STRN3 and SOS1), the radiation-induced alternative transcripts were similar for NSC11 and 0923 cells. For polysome-bound UBE2G2, a change was detected in 0923 but in the opposite direction as that in NSC11. For eIF4H and NDEL1, whereas the changes in the radiation-induced splice events in the polysome fraction were similar between the 2 cell lines, the relative changes detected in the transcriptome (total RNA) were slightly different. Specifically, the eIF4H splice event was significantly increased in 0923 cells, whereas in NSC11 it only trended towards an increase and the NDEL1 splice event was significantly increased in irradiated NSC11 cells but only trended toward an increase in 0923. These results suggest that at least some radiation-induced changes in AS are not unique to NSC11 cells.

### Functional analysis of alternative transcripts detected after irradiation

**Figure 4:**
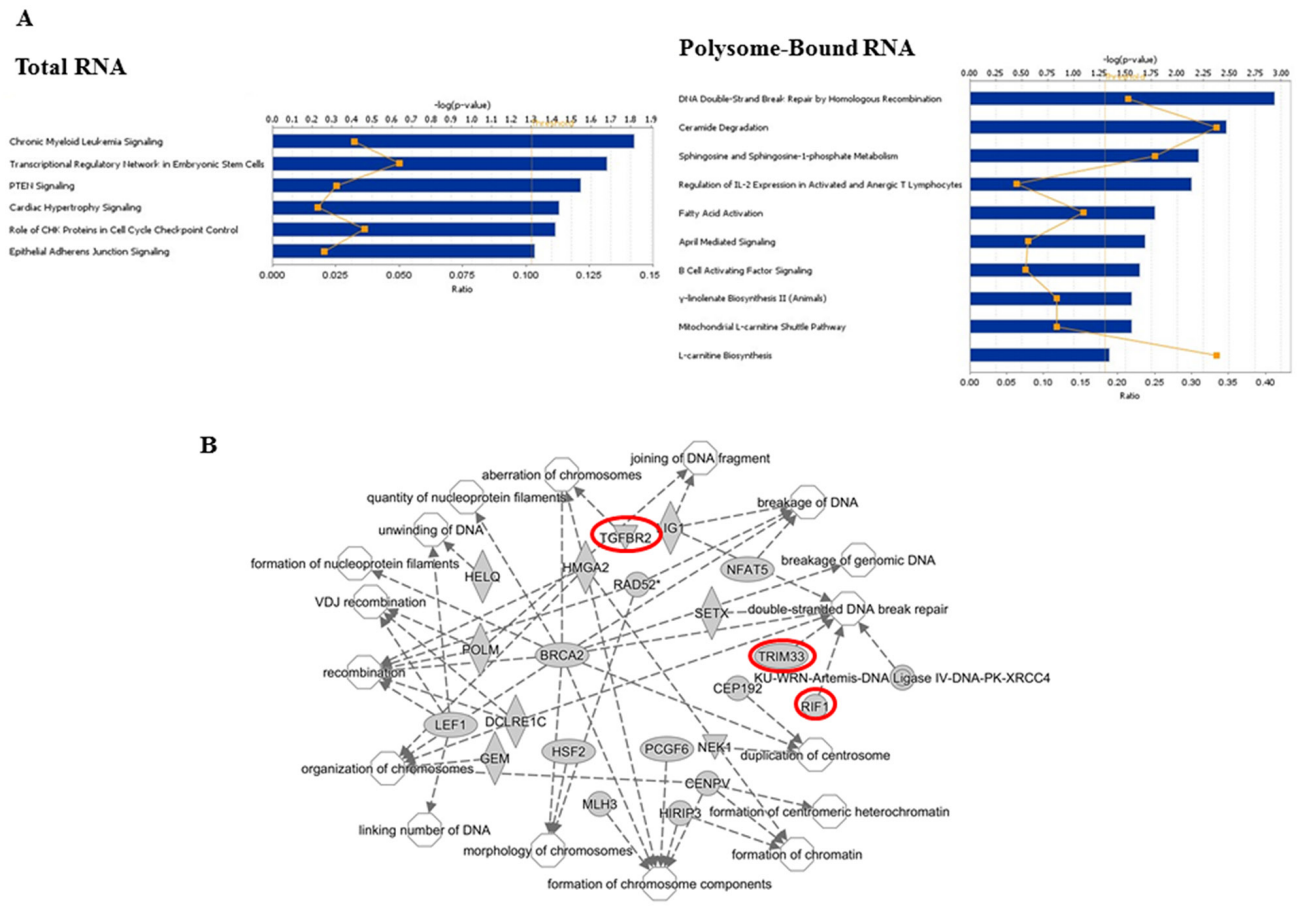
Functional analyses of radiation-induced alternative transcripts **(A)** Canonical pathways as defined by IPA for genes associated with radiation-induced changes in alternative transcripts in total RNA and polysome-bound RNA. Bars correspond to -log(*p*-value) of the pathway’s enrichment. The threshold (dotted orange line) is set by IPA at *p* < 0.05; the p-value of <0.01 equals -log(p-value) of 2. The orange squares indicate the ratio of the number of genes within the pathway affected by radiation out of the total number of genes in the pathway. **(B)** Network analysis of the function *DNA Replication, Recombination, and Repair* showing 22 genes within the network (grey symbols) and the associated 17 subnetworks (unfilled symbols). Symbol shape represent the function of the protein (ellipses, transcriptional regulators; diamonds, enzymes; concentric circles, complexes; triangles, kinases; circles, other). Red highlighted genes are also in the Total RNA group.

The radiation-induced splice events in total cellular RNA (92) mapped to 89 genes and the radiation-induced splice events in polysome-bound RNA (280) mapped to 262 genes (Supplementary Tables 3 and 4). To generate insight into the functional consequences of the radiation-induced alternative transcripts, the gene lists from the total RNA and polysome-bound RNA were uploaded to Ingenuity Pathway Analysis (IPA), which distributes genes into curated networks and associates the networks with cell functions as well as canonical pathways. As shown in Figure 4A, the genes containing the radiation-induced splice events in total RNA distributed to six canonical pathways, none of which were significant at p<0.01 (-log(p-value) ≥ 2). However, the genes containing the radiation-induced splice events in polysome-bound RNA contained 10 canonical pathways, 4 of which were significant (p<0.01): *DNA Double-Strand Break Repair by Homologous Recombination, Ceramide Degradation, Sphingosine and Sphingosine-1-phosphate Metabolism and L-carnitine Biosynthesis*. The networks enriched in total RNA and polysome-bound RNA after irradiation are shown in Tables 1 and 2, respectively. While 9 networks were identified in total RNA, 22 were found in polysome-bound RNA including “*DNA Replication, Recombination, and Repair*”, processes critical in determining cellular radioresponse. This network, which is diagramed in Figure 4B, has 17 subgroups with increased functional specificity (unfilled symbols). In polysome-bound RNA there were 22 genes changed after irradiation (grey symbols, Figure 4B) that map to these specific functions; 3 of these genes (highlighted in red) were also affected at the level of total RNA. These data suggest that the radiation-induced alternative transcripts detected in the polysome bound RNA may contribute to DNA damage response after irradiation.

**Table 1:** Top networks (IPA) enriched in the radiation induced alternative splice events in total RNA [score = -log_10_(p-value)]

Score	Top Networks
40	Cell-To-Cell Signaling and Interaction, Hematological System Development and Function, Inflammatory Response
30	Developmental Disorder, Hereditary Disorder, Metabolic Disease
28	Cell Signaling, Developmental Disorder, Hereditary Disorder
25	Cancer, Endocrine System Disorders, Gastrointestinal Disease
24	Cell-To-Cell Signaling and Interaction, Inflammatory Response, Cellular Growth and Proliferation
23	Cell Morphology, Cellular Function and Maintenance
16	Amino Acid Metabolism, Molecular Transport, Small Molecule Biochemistry
12	Cell Morphology, Cellular Function and Maintenance, Cellular Compromise
2	Cancer

**Table 2:** Top networks (IPA) enriched in the radiation-induced alternative splice events in polysome-bound RNA [score = -log_10_(p-value)]

Score	Top Networks
50	Cell Morphology, Cellular Assembly and Organization, RNA Post-Transcriptional Modification
37	Cancer, Gastrointestinal Disease, Neurological Disease
35	Auditory Disease, Cell Morphology, Cellular Function and Maintenance
32	Cell Morphology, Ophthalmic Disease, Connective Tissue Development and Function
25	Lipid Metabolism, Small Molecule Biochemistry, Hereditary Disorder
25	Post-Translational Modification, Developmental Disorder, Hereditary Disorder
23	Cellular Assembly and Organization, Cellular Function and Maintenance, Developmental Disorder
22	Cellular Growth and Proliferation, Connective Tissue Development and Function, Cell Cycle
21	Cellular Movement, Connective Tissue Disorders, Developmental Disorder
21	DNA Replication, Recombination, and Repair, Energy Production, Nucleic Acid Metabolism
21	Cell Morphology, Organ Morphology, Reproductive System Development and Function
19	Cell Death and Survival, Cellular Compromise, Organ Morphology
18	Cellular Growth and Proliferation, Cell Cycle, Cell-To-Cell Signaling and Interaction
17	Cell Cycle, Tissue Morphology, Cellular Assembly and Organization
17	Auditory Disease, Hereditary Disorder, Neurological Disease
17	Molecular Transport, Small Molecule Biochemistry, Gene Expression
15	Cancer, Developmental Disorder, Hereditary Disorder
15	Cell-To-Cell Signaling and Interaction, Metabolic Disease, Cardiac Enlargement
7	Humoral Immune Response, Protein Synthesis, Cancer
6	Hematological System Development and Function, Tissue Morphology, Cell-To-Cell Signaling and Interaction
2	Cancer, Organismal Injury and Abnormalities, Reproductive System Disease
2	Neurological Disease, Hematological System Development and Function, Organismal Development

### Relationship between radiation-induced AS and gene expression

**Figure 5:**
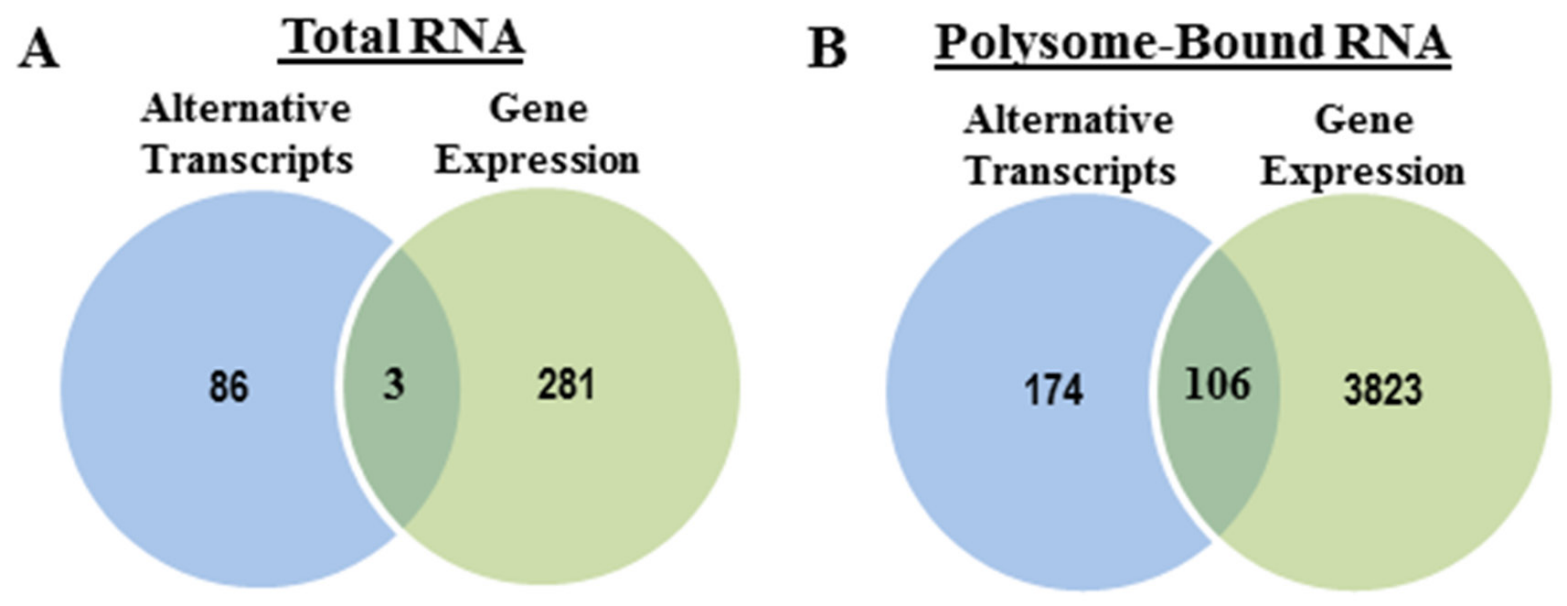
Radiation-induced splice events versus radiation-induced changes in gene expression Venn diagrams comparing the genes with significant splice events after radiation and genes whose overall abundance (expression) was significantly changed after radiation in **(A)** total RNA and **(B)** polysome-bound RNA.

Because RNA-Seq data allows for not only evaluation of alternative transcripts (SpliceSeq), but also changes in gene expression, we compared radiation-induced alternative transcripts to radiation-induced gene expression as detected in total RNA and polysome-bound RNA. In terms of the radiation-induced gene expression substantially more genes were affected by radiation in the translatome (3929) versus the transcriptome (284) (Figure 5), consistent with previous reports based on microarray analysis [8, 9, 24]. In the transcriptome (Figure 5A), comparison of radiation-induced alternative transcripts to gene expression showed little overlap with about 3% of alternative transcripts also being reflected as changes at the gene level. These data suggest that radiation-induced AS of pre-mRNA does not, for the most part, play a role in determining the radiation-induced transcriptome at 1h. However, in the radiation-induced translatome, about 38% of the induced alternative transcripts corresponded to genes whose polysome-association was affected after irradiation (Figure 5B). These data suggest that a portion of the radiation-induced AT enriched in the polysome-bound RNA may directly influence the translational efficiency of the corresponding gene. Functional analysis of the radiation-induced AT detected in the transcriptome and translatome are shown in Figure 4. Regarding the gene expression changes induced in the transcriptome and translatome at 1h after irradiation, results from IPA are shown in Tables 3 and 4 with the molecules in the top 10 networks in the transcriptome and in the translatome listed in Supplementary Tables 5 and 6, respectively. Analyses of the radiation-induced gene expression in total RNA (Table 3) and polysome-bound RNA (Table 4) indicate that networks associated with DNA replication, recombination, and repair, are enriched in both the transcriptome and the translatome.

**Table 3:** Top networks (IPA) enriched in the radiation induced gene expression in total RNA [score = -log_10_(p-value)]

Score	Top Networks
**39**	Cell Death and Survival, Nervous System Development and Function, Tissue Morphology
**32**	Cell Cycle, DNA Replication, Recombination, and Repair, Organismal Development
**27**	Cell Morphology, Organ Morphology, Skeletal and Muscular System Development and Function
**26**	Cell-To-Cell Signaling and Interaction, Inflammatory Response, Cellular Function and Maintenance
**26**	Nervous System Development and Function, Cell Death and Survival, Cellular Compromise
**20**	Reproductive System Development and Function, Nucleic Acid Metabolism, Small Molecule Biochemistry
**20**	Auditory and Vestibular System Development and Function, Embryonic Development, Organ Development
**19**	Cell-To-Cell Signaling and Interaction, Cellular Compromise, Cellular Function and Maintenance
**17**	Cell Death and Survival, Gene Expression, Cell-To-Cell Signaling and Interaction
**13**	Hematological System Development and Function, Hypersensitivity Response, Tissue Morphology
**11**	Hereditary Disorder, Nervous System Development and Function, Neurological Disease
**2**	Developmental Disorder, Hereditary Disorder, Neurological Disease
**2**	Cancer, Neurological Disease, Organismal Injury and Abnormalities
**2**	Lipid Metabolism, Small Molecule Biochemistry, Vitamin and Mineral Metabolism
**2**	Endocrine System Disorders, Hereditary Disorder, Organismal Injury and Abnormalities
**2**	Reproductive System Development and Function, Cellular Movement, Post-Translational Modification
**2**	Cancer, Endocrine System Disorders, Organismal Injury and Abnormalities
**2**	Developmental Disorder, Hereditary Disorder, Ophthalmic Disease

**Table 4:** Top networks (IPA) enriched in the radiation induced gene expression in polysome-bound RNA [score = -log_10_(p-value)]

Score	Top Diseases and Functions
**45**	Developmental Disorder, Hereditary Disorder, Metabolic Disease
**43**	Auditory Disease, Developmental Disorder, Endocrine System Disorders
**43**	Cellular Assembly and Organization, DNA Replication, Recombination, and Repair, Developmental Disorder
**40**	Cellular Assembly and Organization, Cellular Function and Maintenance, Cell Cycle
**40**	Cell Cycle, DNA Replication, Recombination, and Repair, Reproductive System Development and Function
**38**	Cardiovascular Disease, Developmental Disorder, Hematological Disease
**38**	Cellular Function and Maintenance, Nervous System Development and Function, Cell Death and Survival
**38**	Post-Translational Modification, Protein Folding, Lipid Metabolism
**37**	Drug Metabolism, Endocrine System Development and Function, Lipid Metabolism
**36**	Energy Production, Nucleic Acid Metabolism, Small Molecule Biochemistry
**34**	Cancer, Hematological Disease, Immunological Disease
**32**	Cellular Assembly and Organization, Developmental Disorder, Hereditary Disorder
**32**	RNA Post-Transcriptional Modification, Inflammatory Response, Cell Cycle
**32**	Developmental Disorder, Hereditary Disorder, Metabolic Disease
**30**	Auditory Disease, Cardiovascular System Development and Function, Cell-To-Cell Signaling and Interaction
**30**	Hereditary Disorder, Neurological Disease, Psychological Disorders
**28**	Post-Translational Modification, DNA Replication, Recombination, and Repair, Nucleic Acid Metabolism
**28**	Developmental Disorder, Endocrine System Disorders, Hereditary Disorder
**26**	Dermatological Diseases and Conditions, Developmental Disorder, Hereditary Disorder
**26**	Developmental Disorder, Neurological Disease, Organismal Injury and Abnormalities
**26**	Molecular Transport, RNA Trafficking, Cell Cycle
**26**	Embryonic Development, Organ Development, Organ Morphology
**25**	Cell Cycle, Cellular Assembly and Organization, Cellular Function and Maintenance
**25**	Cellular Development, Cellular Function and Maintenance, Cellular Growth and Proliferation
**24**	Gene Expression, Protein Synthesis, Energy Production
**23**	Drug Metabolism, Protein Synthesis, Free Radical Scavenging
**23**	Cellular Assembly and Organization, Cellular Function and Maintenance, Cell Signaling
**21**	Cellular Development, Cell Cycle, Connective Tissue Development and Function
**21**	Molecular Transport, Cell Death and Survival, Cellular Compromise
**21**	Cancer, Hematological Disease, Organismal Injury and Abnormalities
**20**	Respiratory Disease, Organismal Injury and Abnormalities, Renal and Urological Disease
**19**	Developmental Disorder, Hereditary Disorder, Metabolic Disease
**18**	Cell Death and Survival, Carbohydrate Metabolism, Lipid Metabolism
**18**	Gene Expression, Cellular Development, Cancer
**18**	Cell Morphology, Cell Cycle, Nervous System Development and Function
**18**	Connective Tissue Disorders, Developmental Disorder, Hematological Disease
**17**	Cell-To-Cell Signaling and Interaction, Cancer, Organismal Injury and Abnormalities
**17**	Tissue Morphology, Metabolic Disease, Cancer
**17**	Behavior, Cardiovascular System Development and Function, Organ Morphology
**15**	Cellular Development, Hematological System Development and Function, Hematopoiesis

## DISCUSSION

In the study reported here we used RNA-Seq and the mRNA isoform identification tool SpliceSeq to investigate the contribution of AS to radiation-induced gene expression. At the whole genome level, the effects of radiation on AS have previously been reported for human lymphoblasts, fibroblasts [21] and peripheral blood mononuclear cells [20] using exon specific microarray platforms. These studies, conducted on the transcriptome (total cellular mRNA), identified a number of genes (depending on the splicing algorithm applied) subject to radiation-induced alternative splicing. As shown here, transcriptome analysis using RNA-Seq/SpliceSeq identified 92 splice events corresponding to 89 genes at 1h after irradiation of NSC11 cells. Whereas a quantitative comparison to previous studies is not feasible given the different methods, cell types and irradiation protocols, the data presented here further support the induction of AS of pre-mRNA by ionizing radiation. In addition to using SpliceSeq to identify alternative transcripts, RNA-Seq results were analyzed using the sRAP package to define differential gene expression, i.e., changes in transcript abundance. Comparison of these 2 parameters within the radiation-induced transcriptome indicated that the vast majority of alternative transcripts detected (Figure 5) did not correspond to genes whose expression was modified, in contrast to the data generated using exon arrays [20, 21]. These results suggest that, at least in NSC11 cells at 1h after exposure to 2 Gy, radiation-induced AS and radiation-induced gene expression as determined in total cellular mRNA are independent events. Of note, at 1h after irradiation of NSC11 cells most of the radiation-induced changes in the translatome have occurred, whereas the changes in the transcriptome occur at later time points such as 6h [6, 8, 9]. Thus, evaluating later times points after irradiation of NSC11 cells may reveal additional AS events within the transcriptome that as shown in previous studies may participate in the DNA damage response [20-22]. As eluded to earlier, the rationale for evaluating AS events at the early time point of 1h after irradiation was that it reduced the complicating influence of transcriptional changes and allowed for a more direct investigation of the role of AS in radiation-induced translational control of gene expression. Of note, more than half of the AS events detected in both the radiation-induced transcriptome and the radiation-induced translatome were exon skip events, which have been implicated as a source of protein diversity and thus may reflect a change in protein function [13].

Whereas radiation-induced changes in the transcriptome may provide an indicator of radiation exposure [20, 21], it is the radiation-induced translatome that corresponds to changes in protein levels playing a functional role in cellular radioresponse [9]. Thus, given the significance of translational control in radiation-induced gene expression along with reports suggesting that AS influences translational control [25-27], we defined radiation-induced splicing events in polysome-bound RNA. As compared to the transcriptome, substantially more radiation-induced splice events were identified in the translatome and, importantly, the vast majority were unique to the translatome (Figure 2A). This comparison suggests that the AS of pre-mRNA induced by radiation as detected in the transcriptome does not play a significant role in translational control of gene expression but that radiation modifies the polysome association of existing alternative transcripts. Functional analysis of these alternative transcripts with respect to cellular processes and pathways showed that the radiation-induced changes detected in polysome bound RNA were enriched with genes corresponding to DNA repair and other aspects of the DNA damage response (Figure 4, Table 2). These results suggest that, in contrast to the alternative transcripts in the radiation-induced transcriptome for which there were no pathways (Figure 4) and few networks (Table 1) detected relevant to radioresponse, the alternate transcripts in the radiation-induced translatome were associated with processes that can influence cell survival.

An additional difference between the alternative transcripts in the radiation-induced transcriptome and translatome involved the relationship to gene expression. In contrast to the transcriptome, a substantial percentage of the alternative transcripts detected in the radiation-induced translatome corresponded to genes whose expression was also modified, which suggests that the selective polysome association of the existing alternative transcripts has a quantitative effect on radiation-induced gene translation. The alternative transcripts in the radiation-induced translatome that do not overlap with gene expression, while perhaps not affecting translational efficiency, could qualitatively influence other aspects of protein function such as location and activity. With respect to the mechanism through which alternative transcripts are recruited to and away from polysomes, Sterne-Weiler et al. proposed that AS not only provides a process for increasing protein diversity, but also modifies the cis-regulatory structure of mRNAs resulting in an altered susceptibility to post-transcriptional regulatory processes [15]. This scenario could be applicable to the post-transcriptional operon model of Keene and Tenenbaum [28], which proposes that RNA-binding proteins and microRNAs regulate the translation of functionally related genes. Extending these hypotheses to irradiated cells, the AS events operative in untreated NSC11 cells produce a percentage of alternative transcripts for a given gene that have a structural modification that influences its interaction with a radiation-induced RNA-binding proteins (RBP) or microRNA, which then ultimately affects its translation initiation and polysome association. Futhermore, it has been shown that long non-coding RNAs (lncRNAs) also associate with ribosomes and may contribute to the radiation-induced translatome [29]. With respect to specific signaling molecules, ATM, an apical kinase in the radiation-induced DNA damage response, has been implicated in splicesome regulation suggesting a possible role in radiation-induced AS detected in the transcriptome [30]. Whether it plays a role in the radiation-induced recruitment of mRNA isoforms remains to be determined. Clearly, further investigations into the processes through which AS influences translation initiation are required.

The data presented here suggest that whereas radiation induces AS, it is the alternative transcripts present at the time of irradiation that may play a role radiation-induced translational control of gene expression and thus cellular radioresponse. Although the studies described here were conducted using a single human tumor cell line, given that radiation-induced translational control of gene expression is a fundamental component of cellular radioresponse, and that many of the changes in isoforms were also observed in another GSC line, the selective modification of the polysome-binding of existing alternative transcripts is likely to be a common response to radiation. This is supported by the data in Figure 3 showing similar radiation-induced splicing events in another GSC line. Whether there is a difference between tumor and normal cells, as previously shown for radiation-induced translatomes [24] remains to be determined. However, aberrant splicing of pre-mRNA has been implicated in oncogenesis and tumor cell proliferation [31, 32]. Moreover, modulating AS through inhibition of spliceosome activity has been suggested as a cancer treatment strategy [33] with the spliceosome inhibitor E7107 entered into clinical trials [34]. Although more investigation is required, the data presented here indicates the AS plays a role in radiation-induced translational control of gene expression and suggests that the spliceosome may also provide a target for radiosensensitization.

## MATERIALS AND METHODS

### Cell lines and treatments

Studies were performed using the glioblastoma stem-like cell (GSC) lines NSC11 (kindly provided by Dr. Frederick Lang, MD Anderson Cancer Center) and 0923 [35], which were generated from a glioblastoma surgical specimens as previously described [36]. Cell lines were revived every 2 months from frozen stocks made after receiving cell lines and were most recently authenticated in May 2015, by STR analysis (Idexx Laboratories). Neurospheres were maintained in stem cell medium consisting of DMEM/F12 (Invitrogen), B27 supplement (Invitrogen), and human recombinant bFGF and EGF (50 ng/ml each, R&D Systems) at 37°C, 5%CO_2_/5%O_2_. CD133+ NSC11 cells were isolated from neurosphere cultures by FACS and used as a source for the described experiments [37]. The CD133+ cell cultures met the criteria for tumor stem-like cells including self-renewal, differentiation along glial and neuronal pathways, expression of stem cell related genes, and formation of brain tumors when implanted in immunodeficient mice. For use in an experiment, CD133+ neurosphere cultures were disaggregated into single cells as described [37] and seeded onto poly-L-ornithine (Invitrogen)/laminin (Sigma) coated tissue culture dishes in stem cell media. Under these conditions, single-cell GSCs attach and proliferate maintaining their CD133+ expression and stem-like characteristics [38]. All cells were cultured for less than 2 months after resuscitation. Radiation was delivered using a 320 kV X-ray machine (Precision X Ray Inc.) at a dose rate of 2.3 Gy/min; control cultures were mock irradiated.

### Polysome isolation

Isolation of polysome-bound RNA was performed on 6 biological replicates initiated from different frozen stocks following the procedure described by Galban et al [39] with slight modifications. Briefly, cells were grown to ∼80% confluency in 150-mm^2^ culture dishes and irradiated (2 Gy) or mock irradiated. One hour after irradiation cells were incubated with 100 μg/ml of cycloheximide for 15 minutes at 37°C, 5%CO_2_/5%O_2_. Cytoplasmic RNA was collected by lysing cells in polysome buffer [15 mmol/L Tris-HCl (pH 7.5), 300 mmol/L NaCl, 15 mmol/L MgCl2, 1% Triton X100, 100 μg/mL cycloheximide, 1 mg/mL heparin, and 500 units/mL RNasin (Promega)]. After 15 minutes on ice, lysates were centrifuged (12,000 × g for 15 minutes), and the resulting cytosolic supernatant was layered onto a 10% to 50% sucrose gradient. Gradients were then centrifuged at 35,000 × g for 3 hours at 4°C and polysome-bound fractions were collected using an ISCO Density Gradient Fractionation System (ISCO, Lincoln, NE) with continuous monitoring based on A254. Polysome-bound RNA was collected corresponding to pooled fractions 4-12 obtained from sucrose gradient fractionation [9] and extracted using TRIzol LS (Invitrogen). The integrity of the RNA was assured using a Bioanalyzer (Agilent).

### Total RNA isolation

Total RNA, from 6 biological replicates corresponding to the same replicates of polysome-bound RNA, was extracted from irradiated (2 Gy) or mock irradiated cells using TRIzol (Invitrogen) followed by the RNeasy Mini Kit (Qiagen). The integrity of the RNA was assured using a Bioanalyzer (Agilent).

### RNA-seq

RNA-Seq was performed by the Center for Cancer Research Sequencing Facility in Frederick, Maryland. Briefly, between 100ng to 1μg of total RNA was used as the input for mRNA capture with oligo-dT coated magnetic beads. The mRNA was fragmented followed by random-primed cDNA synthesis. The resulting double-strand cDNA was used as the input to a standard Illumina library prep with end-repair, adapter ligation and PCR amplification to generate a sequencing ready library. The final library was then quantitated by qPCR before cluster generation and sequencing on the Illumina HiSeq2500 sequencer. The HiSeq Real Time Analysis software (RTA 1.18) was used for processing image files, the Illumina CASAVA_v1.8.4 was used for demultiplex and converting binary base calls and qualities to fastq format. The sequencing reads were trimmed of adapters and low quality bases using Trimmomatic (version 0.3), the trimmed reads were aligned to human hg19 reference genome (GRCh37/UCSC hg19) and Ensembl annotation version 70 using TopHat_v2.0.8 software. Given the aligned sequencing reads and a list of genomic features, counts of mapped reads for each gene were calculated using HTSeq [40]. (See Supplementary Table 1 and Supplementary Figure 1 for library complexity)

### Alternative transcript and gene expression analysis

The bioinformatics program SpliceSeq, which was developed for alternative splicing analysis of RNA-Seq data [41], was used to identify differential splicing events in polysome-bound RNA and total RNA. SpliceSeq returns gene reads per kilobase of transcript per million aligned reads (RPKM) and percent spliced in (PSI) values for each gene and every potential splice event with sufficient read coverage. If 2 or more reads overlap into adjacent exons by more than 4 bases, it is considered an observed splice junction. The PSI values of 6 irradiated samples were compared to the PSI values of 6 control samples to identify robust splicing events. A splice event was defined as significant according to the following criteria: 1) The event was reproducible in at least 5 of 6 replicates; 2) the gene expression RPKM (measure of general read coverage) ≥ 2; 3) the absolute value of the change in percent of all isoforms displaying the splice event (change in percent spliced in, dPSI) was ≥ 0.1; 4) the proportion of isoforms containing the splice event was ≥ 0.5, and 5) the *p*-value of a t-test for difference between the test and control PSI values was ≤ 0.05.

For gene expression analysis, RPKM reads were used for pairwise comparison between untreated control and irradiated groups for polysome-bound RNA or total RNA samples. This analysis was performed in R [42] with the sRAP package analysis pipeline [43]. Briefly, RPKM values were filtered based upon RPKM cutoff of 0.1 (to avoid bias from low-coverage genes as suggested by http://bioinfo.aizeonpublishers.net/content/2013/6/bioinfo285-292.pdf), and then data is log2 transformed (so that the data more closely follows a normal distribution [43]. In all cases, p-values are calculated via linear regression of ANOVA, and false-discovery rates (FDR) are calculated [44]. We set the p-value and FDR threshold to 0.05. Sequence data has been deposited in NCBI’s Gene Expression Omnibus [45] and are accessible through GEO Series accession number GSE94693 (http://www.ncbi.nlm.nih.gov/geo/query/acc.cgi?acc=GSE94693).

### qPCR of alternative transcripts

Complementary first-strand DNA was generated from irradiated and mock-irradiated total and polysome-bound RNA isolated separately from the RNA used for RNA-Seq, using the Applied Biosystems High-Capacity RNA-to-cDNA Kit (Applied Biosystems, ThermoFisher) according to the manufacturer’s protocol. TaqMan Primers and probes for the specified genes (Applied Biosystems, ThermoFisher), selected based on sequence homology to exon flanking regions, were used and PCR was performed using TaqMan RT-PCR kits (Applied Biosystems, ThermoFisher), according to the manufacturer’s protocol. All assays (probes and primers) used are listed in Supplementary Table 7. Relative fold changes were determined by the ΔΔC_t_ method using 18s (Applied Biosystems, ThermoFisher) as an internal control. Statistical significance (*p* ≤ 0.05) was determined using Student’s *t*-test with data expressed as the mean ± standard error.

### Ingenuity pathway analysis

Genes exhibiting significant changes in alternative splicing or gene expression were submitted to Ingenuity Pathway Analysis (IPA^®^, QIAGEN Redwood City, www.qiagen.com/ingenuity) for core expression analysis and evaluated using canonical pathway and network analyses with IPA default settings and a p-value ≤ 0.01.

## SUPPLEMENTARY MATERIALS FIGURE AND TABLES










