# Supplementary material for: Radiation-induced alternative transcripts as detected in total and polysome-bound mRNA
Source: Oncotarget. 2017 Oct 9;9(1):691–705. doi: 10.18632/oncotarget.21672 (PMC5787501; doi:10.18632/oncotarget.21672)
Supplement: Supplementary file 1 [file oncotarget-09-691-s001.pdf]

Radiation-induced alternative transcripts as detected in total and polysome-bound mRNA

SUPPLEMENTARY MATERIALS

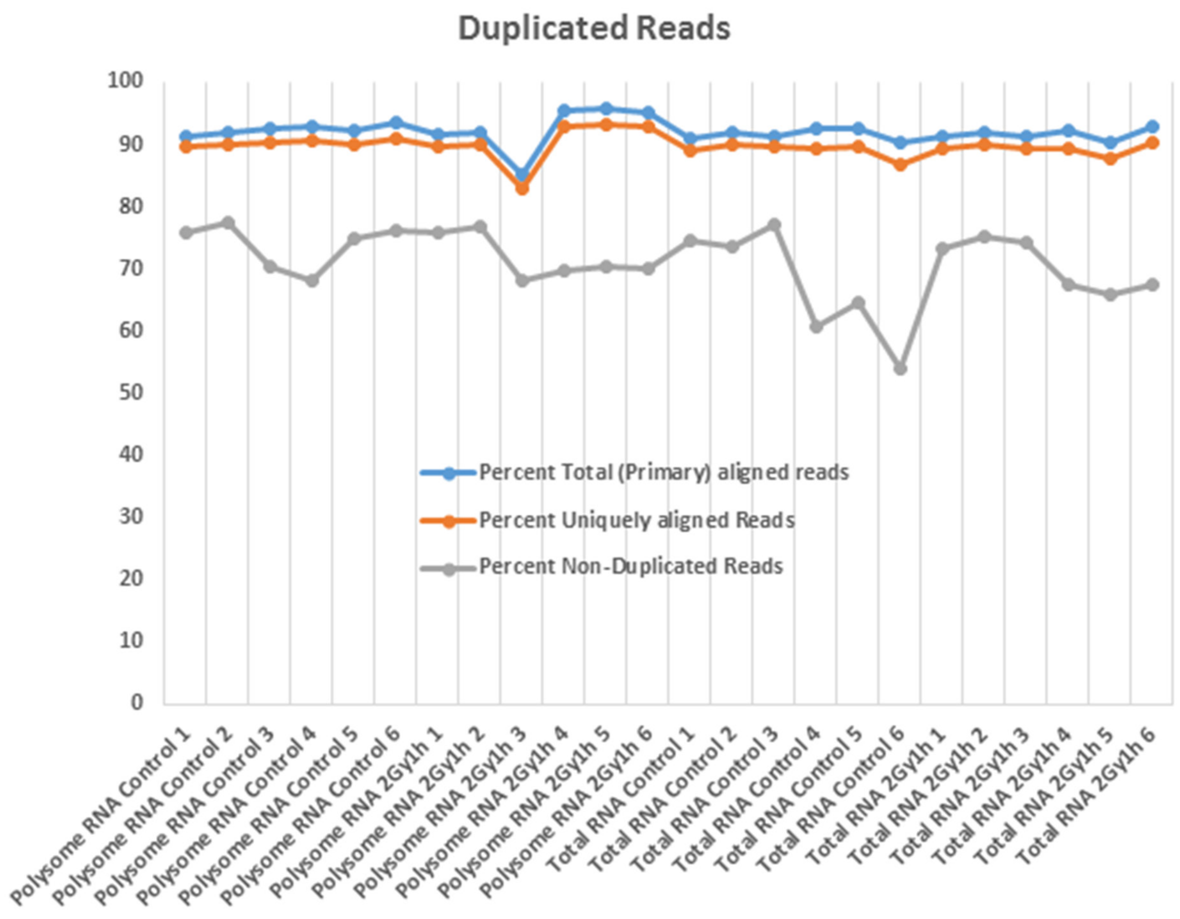

Supplementary Figure 1: Summary of Illumina library complexity.

Supplementary Table 1: Summary of Illumina RNA-Seq read counts.

See Supplementary File 1

Supplementary Table 2: Alternative splice events in the untreated translatome as compared to transcriptome.

See Supplementary File 2

Supplementary Table 3: Alternative splice events in the transcriptome after radiation.

See Supplementary File 3

Supplementary Table 4: Alternative splice events in the translatome after radiation.

See Supplementary File 4

Supplementary Table 5: List of molecules in the top 10 networks in the radiation-induced transcriptome

	Molecules in Network	Top Diseases and Functions
1	14-3-3, Akt, BCR (complex), **CMPK2, CNTN1, EGR2, EGR3, GDNF,** Hsp90, **ID2,** IFN type 1, **IL17RB, IL22RA1,** Immunoglobulin, **IRF5, KIT, LHX3, LIF, LUZP1,** MAP2K1/2, Notch, **NUAK1,** p70 S6k, **PDGF BB, PDGFD, PIM1,** Plk, **PLK2, PLK3,** PP2A, **PRAP1, PRDM1,** PRKAA, **SNAI1,** STAT5a/b	Cell Death and Survival, Nervous System Development and Function, Tissue Morphology
2	**AMER1, ANKRD2,** APC (complex), **AXIN2, BTG2, CCNE1, Cdc2, CDC6, CDC25A, CDCA7L,** Cdk, CDK2-CyclinE, Cyclin A, Cyclin A/Cdk2, Cyclin E, E2f, **E2F6, ENC1,** ERK1/2, **GPC5, HABP2, HAS3,** Hedgehog, **IL12A,** N-cor, **NRIP2, ORC1,** Rb, **ROR2, RRM2,** Rxr, Smad2/3-Smad4, thymidine kinase, VitaminD3-VDR-RXR, Wnt	Cell Cycle, DNA Replication, Recombination, and Repair, Organismal Development
3	26s Proteasome, Actin, Alp, Alpha catenin, AMPK, **ANKS1B,** ATPase, caspase, **CDK2, CDKN1A, CHD1L,** Ck2, cytochrome C, estrogen receptor, **FGF17,** Hdac, HISTONE, Histone H1, Histone h3, Histone h4**, HSPA5, Jnk, JUP, KLF4, PCDH10, PHLDA3,** RNA polymerase II, **SESN1, SESN2, SYPL2, TAF1L,** TCR, **TNNT2, TP53INP1,** Ubiquitin	Cell Morphology, Organ Morphology, Skeletal and Muscular System Development and Function
4	ADCY, **ADORA3,** ADRB, **AMOTL2, ANGPTL7, ARL4C,** calpain, **CCR1, CD93, CDK5R1,** Cg, Creb, **EDN2, ERK,** Focal adhesion kinase, FSH, **GCC1,** Gpcr, **GPRC5A,** Gsk3, **KEL, KIAA1217,** Lh, **MAFF,** Mmp, **OSBPL10,** p85 (pik3r), Pdgf (complex), Pkc(s), PLC, **S1PR3, SRR,** STAT, TSH, Vegf	Cell-To-Cell Signaling and Interaction, Inflammatory Response, Cellular Function and Maintenance
5	ACY1, **AFG3L2, ANGPTL7,** APP, ARPP19, **C3orf33, CCDC115, CDS1,** CINP, **CYB5B,** DMKN, FAM214B, FGF16, IL13, L-dopa, L-glutamic acid, **MAP3K7CL,** NDUFS4, PAIP2, POR, PROK2, **RAD51B, RASL11B, RTP4,** SCN2B, SCUBE3, **SLC25A17,** TMCC2, **TMEM97, TMEM199, TNR, ULK4,** VEGFA, **ZBTB34,** ZFP36	Nervous System Development and Function, Cell Death and Survival, Cellular Compromise
6	**ADORA3, APOL6, BBS4,** beta-estradiol, **C1RL, CACNA2D1,** CACNB1, CACNG8, Collagen(s), cyclic AMP, **FECH,** GALR1, GALR2, GHRHR, **GINS3, GPR84,** GPRC5A, **GRM6,** Hba1/Hba2, HCAR3, hemoglobin, HSD17B, IL1B, PANX1, **PDE11A, PDP1,** PTGFR, **RAD1,** RHNO1, SLC14A1, **SMOC1,** SPP1, SSTR3, TH17 Cytokine, TTC8	Reproductive System Development and Function, Nucleic Acid Metabolism, Small Molecule Biochemistry
7	**ADNP2,** AGT, ATG5, **ATG16L2, AUNIP, C17orf82, C1orf198,** CAND1, **COX15,** DCAKD, EED, EFHD2, ELAVL1, **FAM50B,** FBXO21, GBE1, HCN3, HSP90AA1, ICE2, IGKV1-5, **KLHL36, LRIG3,** MRPS16, NAA25, **NRDE2,** Ppp2r5c, PRMT6, RBM5, **SLC10A6, SLC17A3,** TFIP11, TMEM65, **TSPAN6,** uric acid, XPO4	Auditory and Vestibular System Development and Function, Embryonic Development, Organ Development
8	ADGRE1, APOM, biotin, **BZRAP1,** Ca2+, CACNA1S, CALML3, cholesterol, DAPK2, diacylglycerol, **DMC1, FOXN4, GBP5, KCNJ2,** LPPR4, LSR, miR-19b-3p (and other miRNAs w/seed GUGCAAA), **MYLIP,** NPC1, NR4A2, **PDE1B,** phospholipid, PLA2G2D, **PPAPDC1B,** S100A8, S100A11, **SERPINB8, SGMS2,** Srebp, **STARD5,** SYT3, SYT5, tretinoin, VLDLR, **ZNF16**	Cell-To-Cell Signaling and Interaction, Cellular Compromise, Cellular Function and Maintenance
9	**A1CF,** ABHD17A, **AEN,** ATF2, CAMSAP2, cannabinol, CDC20B, CYTH4, ESR1, **FAM124B,** FOS, FXYD6, HNF1A, **HPGDS,** KCTD9, KRT40, **LY6K,** MEMO1, MFI2, RPS6KA4, SCAND1, **SPEF1, SULT4A1,** TBCA, TRIM27, UBE2I, UBE2T, VSX1, **ZKSCAN4,** ZNF24, **ZNF79, ZNF266,** ZNF317, **ZSCAN25,** ZSCAN32	Cell Death and Survival, Gene Expression, Cell-To-Cell Signaling and Interaction
10	Ap1, Calcineurin protein(s), CaMKII, **CKMT2, ELL2, FASTKD5**, Fc gamma receptor, **GADD45A**, Hsp70, IFN Beta, Iga, Ige, IgG, IgG1, Igm, IL1, IL12 (complex), IL12 (family), Interferon alpha, LDL, Mek, MHC Class II (complex), **MNT**, Nfat (family), NFkB (complex), **NFKBIA**, P38 MAPK**, PANX1**, PI3K (family), Pro-inflammatory Cytokine**, SELPLG**, Tgf beta, Tlr**, TRIAP1, TRIM13**	Hematological System Development and Function, Hypersensitivity Response, Tissue Morphology

Gene names in bold were found to be differentially expressed in the RNA-Seq gene expression analysis.

Supplementary Table 6: List of molecules in the top 10 networks in the radiation-induced translatome

	Molecules in Network	Top Diseases and Functions
1	**CGREF1, CIRH1A, CMC1, COA3, COMMD1, COMMD2, COMMD4, COMMD5, COMMD7, COX14, COX4I1, COX6A1, COX6B1, DOHH, GJC1, MRPL46,** NFkB (complex), **NGFRAP1, NMRAL1, PAM16, PKN3, PNKD, RELT, SLC25A6, SLC2A6, TIMM10, TIMM22, TIMM23, TIMM17B, TIMM8A, TMEM223, TMEM14C, TOMM40, WDR34, ZNF71**	Developmental Disorder, Hereditary Disorder, Metabolic Disease
2	**ANAPC5, ANAPC11, ANAPC16, ARMC6, BUB3, C20orf24, CCDC24, CDC23, CLPP, COPS3, COPS6, COPS7A,** E3 RING, **FBXL15, FBXW9, FEM1A, GNG3, GNG10, GPS1, HYAL2, IDH3B, LRR1,** Mapk, **MRPL41, PDCD2L, PHLDA3, PMF1/PMF1-BGLAP, QTRT1, RBX1, SSR3, TCEB2, TCEB3C (includes others), TMEM11, WRAP73, ZC3HC1**	Auditory Disease, Developmental Disorder, Endocrine System Disorders
3	**AP5B1, AP5S1, ASF1B, C19orf25, C8orf48, CARD9, CBX2, CCDC71, CCDC85B, CDCA4,** chemokine, chemokine receptor, **E2F2, ENKD1, FASTK, H3F3A/H3F3B, HIST1H2AB, HIST1H3C, HIST1H3F, IFRD2, KIF9, MCM2, MCM5, MCM7, MLF2, MPDU1, MRPS7, NUP85, ORC6, PDLIM7, PMVK, PSMD2, SLC1A5, TSSC1, ZFAND2A**	Cellular Assembly and Organization, DNA Replication, Recombination, and Repair, Developmental Disorder
4	**ACAP3, ASNA1, BCL2L12, C19orf60, CAK, CCNO,** Cdk, CDK2-CyclinE, **CDKN1A, CLEC3B, CPSF3L, DALRD3, EIF6, FH, FLAD1, GET4, HAUS4, HAUS5, HAUS7, HAUS8, KLF16, MRPL37, MYDGF, NEK8, NOC2L, PCBP4, PHKG2, RNF126, SEC61B, SGTA, TALDO1, TCTEX1D2, TK1, WDR18, WDR73**	Cellular Assembly and Organization, Cellular Function and Maintenance, Cell Cycle
5	**ABT1, ACD, ALG12, BANF1, BSCL2, BYSL, CAPZB, DDX41, EEF1D, EIF5A, EMD,** G protein alphai, **GDPD5, GTPBP8,** HDL-cholesterol, **HSPBP1, KCNQ2, LIMD2, MRPL20, MRPL53, MVK, NEIL2, NHEJ1, NHLRC1, PNKP, PUF60, SARS2, SIAH1, SIRT6, SMO, SOX18, STUB1,** tubulin (complex), **UXT, XRCC6**	Cell Cycle, DNA Replication, Recombination, and Repair, Reproductive System Development and Function
6	**BOP1, CD320, CDCA5,** Cyclin D1/cdk4, **EGFL7, ERK, FAU, FRS3, ILVBL, LOXL1, MAFF, RER1,** Ribosomal 40s subunit, Rnr, **RPAIN, RPS2, RPS3, RPS5, RPS8, RPS10, RPS12, RPS14, RPS15, RPS16, RPS19, RPS21, RPS26, RPS28, RPS29, SIPA1, THEMIS2, TSR2, TXNL4B, UBL4A, UMPS**	Cardiovascular Disease, Developmental Disorder, Hematological Disease
7	**AIMP2, APRT, BLOC1S4, C9orf116,** Caveolin, **DDX28, DDX49, DNPH1, DTNBP1, EIF3K, FHL3, FTSJ1, FTSJ2, GALNT15,** Integrin alpha 5 beta 1, methyltransferase, **MIS18A, MRPL12, MRPL27, MRPL38, MYCBP, NAPA, NOL12, OIP5,** Pka, **PRMT1, RAB32, RALY, RNMTL1, SLC25A10, SNAPIN, SPSB2, TRMT112, TYMSOS, WBSCR22**	Cellular Function and Maintenance, Nervous System Development and Function, Cell Death and Survival
8	APC/APC2, **BCL2L13, C19orf70, CARHSP1, CDK10,** CK1, **CLN3, CMTM6, CRELD2, CYC1, ELOVL1, FAM64A, FKBP1A, FKBP1B,** Gsk3, **HINT2, MOCS1, MRPL14,** peptidylprolyl isomerase, **PIN1, PPIE, PPIF, PREB, SFXN4, SLC25A4, SLC25A11, SLC25A19, SSBP4, TECR, TELO2, TIMM13, TIMM50, TMEM128, TOMM22, TSPO**	Post-Translational Modification, Protein Folding, Lipid Metabolism
9	**ACTL6A, ACTR5, APOA1BP, ASL, CDK20,** cyclooxygenase, **EPDR1, GSS, H2AFX, H2AFZ, HMOX1, HSD11B2, INO80C, INO80E, MAT2A, MCRS1, MYL6B, NTPCR,** Nuclear factor 1, **PITX3, POFUT1, POLR2E, POMC, PROCR, RRP9, RUVBL2, SLC25A5, SNU13,** Stat3-Stat3, **TFPT, TIP60, WDR61, WRAP53, ZNHIT1, ZNHIT2**	Drug Metabolism, Endocrine System Development and Function, Lipid Metabolism
10	adenosine-tetraphosphatase, ATP synthase, **ATP5B**, **ATP5D**, **ATP5G1**, **ATP5G3**, **ATP5S**, **CDK2AP2**, **DERL2**, **DPM2**, **DTYMK**, **EBP**, **EBPL**, **ECSIT**, F0 ATP synthase, **FOXRED1**, **GCDH**, **HSD17B7**, **JAGN1**, **LAMTOR4**, **MCOLN1**, **MSRB1**, **MT1X**, **NID2**, **PEMT**, **PEX16**, **PIGH**, **PKDCC**, **PPA2**, **PYCR1**, **SLC25A1**, **VDAC2**, Vegf, **VKORC1**, **YIF1A**	Energy Production, Nucleic Acid Metabolism, Small Molecule Biochemistry

Gene names in bold were found to be differentially expressed in the RNA-Seq gene expression analysis.

Supplementary Table 7: TaqMan gene expression assays

	TaqMan Assay ID
UBE2G2	Hs01119563_m1
TGFBR2	Hs00947875_m1
STRN3	Hs00923644_m1
eIF4H	Hs01091758_g1
NDE11	Hs00229366_m1
SOS1	Hs00893135_m1
18S	Hs99999901_s1
