# Supplementary material for: Radiation-induced alternative transcripts as detected in total and polysome-bound mRNA
Source: Oncotarget. 2017 Oct 9;9(1):691–705. doi: 10.18632/oncotarget.21672 (PMC5787501; doi:10.18632/oncotarget.21672)
Supplement: Supplementary file 2 [file oncotarget-09-691-s002.docx]

| **Sample ID** | Total RNA Control 1 | Total RNA Control 2 | Total RNA Control 3 | Total RNA Control 4 | Total RNA Control 5 | Total RNA Control 6 | Total RNA 2Gy1h 1 | Total RNA 2Gy1h 2 | Total RNA 2Gy1h 3 | Total RNA 2Gy1h 4 | Total RNA 2Gy1h 5 | Total RNA 2Gy1h 6 |
| --- | --- | --- | --- | --- | --- | --- | --- | --- | --- | --- | --- | --- |
| **Yield (Mbases)** | 19,806 | 16,985 | 21,044 | 20,567 | 19,121 | 18,833 | 18,976 | 20,435 | 18,410 | 17,433 | 17,600 | 19,751 |
| **# Reads** | 196,103,296 | 168,168,136 | 208,352,724 | 203,631,568 | 189,318,756 | 186,461,962 | 187,880,974 | 202,326,758 | 182,279,192 | 172,600,660 | 174,254,756 | 195,556,398 |
| **% of >= Q30 Bases (PF)** | 88.19 | 89.34 | 88.32 | 87.07 | 87.41 | 88.01 | 88.15 | 89.34 | 88.22 | 87.32 | 92.42 | 87.95 |
| **Percent Total (Primary) aligned reads** | 90.84 | 91.90 | 91.35 | 92.62 | 92.71 | 90.25 | 91.22 | 91.79 | 91.20 | 92.21 | 90.45 | 92.97 |
| **Percent Uniquely aligned Reads** | 88.94 | 90.06 | 89.55 | 89.48 | 89.70 | 86.91 | 89.35 | 89.90 | 89.38 | 89.35 | 87.77 | 90.22 |
| **Percent Non-Duplicated Reads** | 74.71 | 73.57 | 77.04 | 60.81 | 64.62 | 54.11 | 73.20 | 75.32 | 74.12 | 67.48 | 65.73 | 67.39 |
| **Percent Usable Bases** | 90.86 | 90.98 | 90.88 | 94.29 | 93.79 | 94.65 | 90.99 | 90.93 | 90.38 | 93.14 | 92.17 | 92.59 |

**Supplementary Table 1: Summary of Illumina RNA-Seq read counts**

| **Sample ID** | Polysome RNA Control 1 | Polysome RNA Control 2 | Polysome RNA Control 3 | Polysome RNA Control 4 | Polysome RNA Control 5 | Polysome RNA Control 6 | Polysome RNA 2Gy1h 1 | Polysome RNA 2Gy1h 2 | Polysome RNA 2Gy1h 3 | Polysome RNA 2Gy1h 4 | Polysome RNA 2Gy1h 5 | Polysome RNA 2Gy1h 6 |
| --- | --- | --- | --- | --- | --- | --- | --- | --- | --- | --- | --- | --- |
| **Yield (Mbases)** | 18,848 | 19,724 | 18,460 | 20,635 | 10,841 | 9,866 | 20,064 | 18,496 | 10,584 | 21,142 | 18,321 | 16,667 |
| **# Reads** | 186,611,282 | 195,290,288 | 182,767,034 | 204,311,820 | 107,338,422 | 97,678,690 | 198,651,730 | 183,131,754 | 104,787,390 | 209,323,268 | 181,394,106 | 165,026,664 |
| **% of >= Q30 Bases (PF)** | 88.62 | 89.12 | 87.86 | 87.84 | 84.54 | 84.93 | 88.57 | 89.06 | 84.37 | 83.1 | 82.73 | 82.94 |
| **Percent Total (Primary) aligned reads** | 91.21 | 91.86 | 92.69 | 92.97 | 92.39 | 93.56 | 91.49 | 91.84 | 85.33 | 95.4 | 95.8 | 95.15 |
| **Percent Uniquely aligned Reads** | 89.61 | 90.16 | 90.27 | 90.55 | 89.92 | 91.11 | 89.72 | 90.13 | 82.78 | 92.81 | 93.26 | 92.75 |
| **Percent Non-Duplicated Reads** | 75.97 | 77.30 | 70.32 | 68.08 | 74.74 | 76.23 | 75.88 | 76.96 | 68.12 | 69.77 | 70.38 | 70.02 |
| **Percent Usable Bases** | 93.67 | 94.53 | 93.38 | 93.68 | 93.50 | 93.39 | 95.35 | 95.32 | 90.36 | 94.14 | 95.30 | 95.12 |
