# Supplementary material for: Radiation-induced alternative transcripts as detected in total and polysome-bound mRNA
Source: Oncotarget. 2017 Oct 9;9(1):691–705. doi: 10.18632/oncotarget.21672 (PMC5787501; doi:10.18632/oncotarget.21672)
Supplement: Supplementary file 3 [file oncotarget-09-691-s003.docx]

**Supplementary Table 2: Alternative splice events in the untreated translatome as compared to the untreated transcriptome**

| Gene Symbol | | Splice Type |  | Gene Symbol | | | Splice Type | |  | | Gene Symbol | | Splice Type |
| --- | --- | --- | --- | --- | --- | --- | --- | --- | --- | --- | --- | --- | --- |
| AARS2 | | ES |  | C14orf159 | | | ES | |  | | CHRNA1 | | ES |
| ABCC5 | | RI |  | C14orf79 | | | AP | |  | | CIRBP | | RI |
| ABCD4 | | ES |  | C16orf13 | | | ES | |  | | CLCC1 | | ES |
| ABHD14B | | RI |  | C16orf58 | | | RI | |  | | CLDN15 | | RI |
| ABHD17A | | AD |  | C16orf91 | | | RI | |  | | CLK1 | | ES |
| ABTB1 | | AP |  | C17orf53 | | | ES | |  | | CLK2 | | ES |
| ACAA1 | | RI |  | C17orf58 | | | RI | |  | | CLK4 | | ES |
| ACOX3 | | ES |  | C19orf48 | | | RI | |  | | CMC2 | | ME |
| ADAM22 | | ES |  | C1orf112 | | | AP | |  | | CNOT4 | | AT |
| ALAS1 | | ES |  | C1orf159 | | | AD | |  | | COQ4 | | RI |
| AMDHD2 | | RI |  | C1orf50 | | | ES | |  | | COX11 | | RI |
| ANGEL1 | | AP |  | C1orf51 | | | AP | |  | | CREB3L4 | | RI |
| ANKRD10 | | AT |  | C1orf54 | | | AP | |  | | CREM | | ES |
| ANKRD49 | | RI |  | C20orf96 | | | AT | |  | | CROT | | AT |
| ANKRD9 | | RI |  | C21orf58 | | | RI | |  | | CSNK1D | | ES |
| ANO6 | | ES |  | C2orf42 | | | ES | |  | | CSPP1 | | ES |
| ANO8 | | AA |  | C2orf68 | | | AT | |  | | CTTNBP2 | | AD |
| APAF1 | | ES |  | C5orf45 | | | RI | |  | | CXorf40A | | AP |
| APTX | | ES |  | C7orf43 | | | AP | |  | | CYB5RL | | ES |
| ARAP1 | | AP |  | C9orf85 | | | AT | |  | | D2HGDH | | ES |
| ARL14EP | | ES |  | CALCOCO1 | | | RI | |  | | DALRD3 | | RI |
| ARL6 | | AT |  | CAPN10 | | | ES | |  | | DCAF8 | | ES |
| ARMC10 | | ES |  | CARKD | | | AA | |  | | DCUN1D4 | | AD |
| ARMC5 | | RI |  | CASK | | | ES | |  | | DDX11 | | RI |
| ARPP19 | | RI |  | CASP3 | | | ES | |  | | DDX17 | | AP |
| ARRDC1 | | RI |  | CASP8 | | | ES | |  | | DENND1A | | AT |
| ASTE1 | | ES |  | CC2D1B | | | RI | |  | | DEPDC1 | | ES |
| ATF7 | | AT |  | CCDC14 | | | RI | |  | | DFFB | | AA |
| ATP5J2 | | AD |  | CCDC24 | | | RI | |  | | DIS3L2 | | RI |
| ATPIF1 | | RI |  | CCDC74A | | | RI | |  | | DIXDC1 | | AT |
| ATRAID | | AD |  | CCDC74B | | | AT | |  | | DMAP1 | | RI |
| B9D1 | | RI |  | CCDC90B | | | AD | |  | | DMPK | | AP |
| BAI2 | | ES |  | CCNL1 | | | ES | |  | | DNAJB6 | | AT |
| BAIAP2 | | AT |  | CCNL2 | | | ES | |  | | DNAJC25 | | ES |
| BCAS3 | | ES |  | CCNT1 | | | ES | |  | | DNAL1 | | ES |
| BCLAF1 | | ES |  | CDK10 | | | RI | |  | | DNMT3B | | ES |
| BICD1 | | AD |  | CDK2 | | | RI | |  | | DOK4 | | RI |
| BOLA3 | | ES |  | CENPT | | | AT | |  | | DTNA | | AT |
| BRSK2 | | AA |  | CEP68 | | | AT | |  | | DTX3 | | RI |
| BSDC1 | | RI |  | CEP95 | | | ES | |  | | EFCAB2 | | ES |
| BTAF1 | | ES |  | CERCAM | | | RI | |  | | EFHC1 | | RI |
| BUB3 | | AA |  | CGREF1 | | | ES | |  | | EIF4H | | ES |
| BUD31 | | AD |  | CHEK1 | | | RI | |  | | ELMOD3 | | RI |
| C11orf68 | | AP |  | CHRD | | | RI | |  | | ENDOV | | RI |
| Gene Symbol | Splice Type |  | | Gene Symbol | Splice Type | |  | | Gene Symbol | | Splice Type | | |
| ENOSF1 | ES |  | | HCFC1R1 | ES | |  | | LUC7L3 | | RI | | |
| EPB41 | ES |  | | HDAC10 | RI | |  | | MAGI2 | | ES | | |
| EPC1 | AP |  | | HDAC4 | AA | |  | | MAP2 | | ES | | |
| EXOC7 | ES |  | | HDAC6 | RI | |  | | MAPK10 | | AA | | |
| EXOSC9 | RI |  | | HDDC2 | ES | |  | | MAPK11 | | RI | | |
| FADS3 | RI |  | | HEATR5B | ES | |  | | MAPK8 | | AA | | |
| FAM111A | AA |  | | HES6 | RI | |  | | MARS | | RI | | |
| FAM120AOS | AP |  | | HEXIM2 | AP | |  | | MAT2A | | AT | | |
| FAM133B | AA |  | | HIPK3 | ES | |  | | MAZ | | ES | | |
| FAM13B | ES |  | | HKR1 | ES | |  | | MBD4 | | AT | | |
| FAM156A | RI |  | | HMBS | RI | |  | | MCAT | | ES | | |
| FAM156B | RI |  | | HNRNPA1L2 | ES | |  | | MECOM | | AA | | |
| FAM161A | ES |  | | HSD11B1L | AT | |  | | MEF2A | | ES | | |
| FAM173A | ES |  | | HUS1 | AD | |  | | METTL17 | | RI | | |
| FAM178A | AT |  | | IDE | ES | |  | | METTL23 | | ES | | |
| FAM185A | AD |  | | IDS | AT | |  | | METTL3 | | RI | | |
| FAM188A | ES |  | | IFT20 | AT | |  | | MFSD10 | | RI | | |
| FAM189A1 | ES |  | | IFT52 | AD | |  | | MIEF2 | | AA | | |
| FAM193B | AD |  | | IFT88 | ES | |  | | MIS12 | | ES | | |
| FAM219A | AD |  | | IMMP1L | ES | |  | | MITD1 | | ES | | |
| FAM219B | ES |  | | ING3 | ES | |  | | MKI67 | | ES | | |
| FAM21C | ES |  | | INTS6 | ES | |  | | MLH3 | | ES | | |
| FAM73B | AD |  | | IP6K2 | RI | |  | | MLTK | | AT | | |
| FANCA | AT |  | | IQCB1 | ES | |  | | MPI | | AT | | |
| FASTK | RI |  | | IQCJ-SCHIP1 | ES | |  | | MRPL22 | | ES | | |
| FBXO44 | ES |  | | IQGAP1 | ES | |  | | MRPS18C | | ES | | |
| FLAD1 | RI |  | | IRF7 | RI | |  | | MRPS24 | | RI | | |
| FMR1 | ES |  | | ISOC2 | ES | |  | | MRS2 | | AT | | |
| FN1 | ES |  | | JMJD7 | RI | |  | | MTERFD3 | | RI | | |
| FNDC3B | AT |  | | KANSL3 | AA | |  | | MTHFD2L | | AT | | |
| FRYL | ES |  | | KCNAB3 | ES | |  | | MTHFS | | AD | | |
| GAS2L1 | RI |  | | KCTD17 | ES | |  | | MTMR10 | | RI | | |
| GBA2 | RI |  | | KCTD7 | AP | |  | | MUTYH | | AA | | |
| GCFC2 | AT |  | | KDM6A | ES | |  | | MXD3 | | RI | | |
| GFOD2 | AT |  | | KIAA0907 | RI | |  | | MZF1 | | AT | | |
| GLI4 | AT |  | | KIF21A | ES | |  | | N6AMT2 | | ES | | |
| GNAS | ES |  | | KIF23 | ES | |  | | NAA16 | | ES | | |
| GOLGA8A | RI |  | | KRBOX4 | AT | |  | | NAA40 | | AA | | |
| GPATCH2L | AT |  | | LEF1 | ES | |  | | NADSYN1 | | AP | | |
| GPRASP2 | ES |  | | LEMD2 | AP | |  | | NASP | | ES | | |
| GRIA3 | ES |  | | LENG8 | RI | |  | | NAT14 | | RI | | |
| GSTK1 | RI |  | | LETMD1 | ES | |  | | NAT6 | | AA | | |
| GTF2H2C | ES |  | | LIN37 | RI | |  | | NAT9 | | RI | | |
| GTPBP3 | RI |  | | LRIF1 | ES | |  | | NBPF10 | | ES | | |
| HAGHL | RI |  | | LRP12 | ES | |  | | NBPF11 | | RI | | |
| HARS2 | RI |  | | LRRC23 | ES | |  | | NCOA5 | | ES | | |
| HAUS5 | RI |  | | LRRC42 | ES | |  | | NCOR2 | | ES | | |
| HAUS7 | ES |  | | LUC7L | AD | |  | | NDUFAF5 | | ES | | |
|  |  |  | |  |  | |  | |  | |  | | |
|  |  |  | |  |  | |  | |  | |  | | |
|  |  |  | |  |  | |  | |  | |  | | |
|  |  |  | |  |  | |  | |  | |  | | |
| Gene Symbol | Splice Type |  | | Gene Symbol | Splice Type | |  | | Gene Symbol | | Splice Type | | |
| NDUFS2 | RI |  | | PIGL | AT | |  | | RALGPS2 | | ES | | |
| NECAB3 | RI |  | | PIGP | AP | |  | | RANGRF | | RI | | |
| NFATC1 | AT |  | | PIGX | AT | |  | | RB1CC1 | | AD | | |
| NFATC2IP | AP |  | | PILRB | RI | |  | | RBM6 | | RI | | |
| NFATC4 | RI |  | | PINLYP | AP | |  | | RC3H2 | | AT | | |
| NFKB2 | AA |  | | PKIG | ES | |  | | RCOR3 | | AT | | |
| NISCH | RI |  | | PKMYT1 | AP | |  | | RECQL5 | | RI | | |
| NMRK1 | ES |  | | PLD2 | RI | |  | | RELL1 | | AT | | |
| NOL12 | RI |  | | PLEKHA1 | AD | |  | | RELL2 | | AD | | |
| NPHP3 | AT |  | | PMPCB | RI | |  | | RFX2 | | ES | | |
| NPRL2 | RI |  | | PNISR | AD | |  | | RGAG4 | | AT | | |
| NR2C2AP | AT |  | | PNRC1 | AP | |  | | RHOT2 | | RI | | |
| NRBP2 | RI |  | | POFUT2 | AD | |  | | RMND1 | | AD | | |
| NSUN5 | RI |  | | POLB | ES | |  | | RMND5B | | AD | | |
| NT5C2 | ES |  | | POLL | RI | |  | | RPS6KA3 | | AA | | |
| NT5M | AD |  | | POLM | RI | |  | | RRNAD1 | | RI | | |
| NUDT18 | RI |  | | POLR2J2 | ES | |  | | RUFY3 | | AT | | |
| NUDT6 | AP |  | | POLR2J3 | AA | |  | | RWDD1 | | ES | | |
| NUPL2 | ES |  | | POLRMT | AD | |  | | RWDD2A | | RI | | |
| OARD1 | ES |  | | POM121 | AT | |  | | S100A2 | | AP | | |
| OGFOD2 | ES |  | | PORCN | AA | |  | | SAFB2 | | AD | | |
| OGFR | AP |  | | POSTN | ES | |  | | SAYSD1 | | AP | | |
| OS9 | ES |  | | PPAPDC1B | AT | |  | | SCAF11 | | AT | | |
| OSBPL3 | AD |  | | PPFIA3 | RI | |  | | SCAMP5 | | ES | | |
| OSBPL6 | ES |  | | PPHLN1 | AT | |  | | SCML2 | | ES | | |
| OSGEP | AP |  | | PPM1M | RI | |  | | SCRN2 | | AA | | |
| OXLD1 | AD |  | | PQBP1 | AD | |  | | SDAD1 | | AA | | |
| P2RX5 | ES |  | | PQLC3 | ES | |  | | SDR39U1 | | ME | | |
| P4HTM | ES |  | | PRMT2 | ES | |  | | SEC14L2 | | AT | | |
| PABPC1L | AP |  | | PRMT7 | ES | |  | | SEC61A2 | | ES | | |
| PABPN1 | RI |  | | PRPF3 | ES | |  | | SEMA6C | | AD | | |
| PANK2 | AD |  | | PRPF39 | ES | |  | | SETD4 | | RI | | |
| PARP2 | RI |  | | PRR19 | RI | |  | | SGSM2 | | RI | | |
| PCGF5 | AT |  | | PSMA1 | AD | |  | | SGSM3 | | RI | | |
| PCMT1 | AT |  | | PSMC3IP | AD | |  | | SHOX2 | | AA | | |
| PCNP | AA |  | | PTPN18 | ES | |  | | SIDT2 | | RI | | |
| PCTP | AA |  | | PTPRS | ES | |  | | SLC15A4 | | ES | | |
| PDCD2 | RI |  | | PUS7 | AD | |  | | SLC25A25 | | AP | | |
| PDE4DIP | AT |  | | PVRL3 | AT | |  | | SLC25A29 | | RI | | |
| PDZD4 | ES |  | | PWWP2A | AT | |  | | SLC26A6 | | AP | | |
| PEX1 | ES |  | | QTRT1 | RI | |  | | SLC29A2 | | AA | | |
| PEX13 | AT |  | | QTRTD1 | ES | |  | | SLC39A13 | | RI | | |
| PFN2 | AA |  | | RAB15 | AD | |  | | SLC9B2 | | AT | | |
| PHF14 | AT |  | | RAB24 | RI | |  | | SLCO4A1 | | RI | | |
| PHF19 | AT |  | | RAD51D | ES | |  | | SLMO1 | | ES | | |
| PHF8 | ES |  | | RAD52 | AT | |  | | SMARCD3 | | RI | | |
| PIDD | RI |  | | RAD54B | AT | |  | | SOD2 | | RI | | |
| PIGG | AD |  | | RALGPS1 | AT | |  | | SPAG16 | | AT | | |
|  |  |  | |  |  | |  | |  | |  | | |
|  |  |  | |  |  | |  | |  | |  | | |
|  |  |  | |  |  | |  | |  | |  | | |
|  |  |  | |  |  | |  | |  | |  | | |
| Gene Symbol | Splice Type |  | | Gene Symbol | Splice Type | |  | | Gene Symbol | | Splice Type | | |
| SPATA24 | AT |  | | TMEM53 | AT | |  | | YTHDC2 | | ES | | |
| SPATA33 | AP |  | | TMEM55B | RI | |  | | ZBED5 | | AA | | |
| SPATA5L1 | AD |  | | TMUB2 | ES | |  | | ZCCHC10 | | AT | | |
| SPEG | AD |  | | TNIK | AT | |  | | ZCCHC8 | | AP | | |
| SPHK1 | AP |  | | TOP3B | RI | |  | | ZDHHC15 | | ES | | |
| SPIN2B | RI |  | | TOR2A | RI | |  | | ZFAND2B | | RI | | |
| SRSF1 | RI |  | | TRA2A | ES | |  | | ZFAND5 | | ES | | |
| SRSF2 | RI |  | | TRA2B | ES | |  | | ZFC3H1 | | RI | | |
| SRSF5 | AA |  | | TREX1 | RI | |  | | ZFP1 | | AP | | |
| SSH1 | ES |  | | TRIM13 | RI | |  | | ZKSCAN5 | | AP | | |
| SSH2 | AT |  | | TRIM36 | AP | |  | | ZNF124 | | AA | | |
| SSSCA1 | RI |  | | TRIM46 | ES | |  | | ZNF211 | | AP | | |
| ST7 | AP |  | | TRMT13 | RI | |  | | ZNF226 | | AA | | |
| ST7L | ES |  | | TRMT44 | AA | |  | | ZNF232 | | AD | | |
| STK16 | AA |  | | TRMU | ES | |  | | ZNF234 | | AD | | |
| STK32C | AP |  | | TROAP | RI | |  | | ZNF273 | | AT | | |
| STX1A | AD |  | | TRPM3 | ES | |  | | ZNF286A | | AA | | |
| SUCO | ES |  | | TRPT1 | ES | |  | | ZNF300 | | RI | | |
| SUGP2 | RI |  | | TTC14 | RI | |  | | ZNF302 | | AD | | |
| SULT1A3 | RI |  | | TTC31 | RI | |  | | ZNF331 | | AP | | |
| SUPT20H | AA |  | | TTC39C | AT | |  | | ZNF333 | | AT | | |
| SUPT7L | RI |  | | TTYH2 | AP | |  | | ZNF43 | | ES | | |
| SYDE1 | RI |  | | TUBE1 | AA | |  | | ZNF506 | | AT | | |
| TADA1 | ES |  | | U2SURP | ES | |  | | ZNF511 | | RI | | |
| TAF1C | RI |  | | UBALD1 | RI | |  | | ZNF524 | | AP | | |
| TAF1D | AA |  | | UBAP2 | ES | |  | | ZNF528 | | AT | | |
| TANK | ES |  | | UBAP2L | AT | |  | | ZNF550 | | ES | | |
| TARBP1 | ES |  | | UBXN11 | AT | |  | | ZNF559 | | AD | | |
| TCEA2 | RI |  | | UBXN4 | RI | |  | | ZNF584 | | ES | | |
| TEFM | RI |  | | ULK3 | RI | |  | | ZNF585A | | AT | | |
| TEX30 | ES |  | | UNC119 | AP | |  | | ZNF599 | | AT | | |
| THTPA | AD |  | | UQCC1 | AT | |  | | ZNF606 | | AT | | |
| THUMPD3 | AD |  | | USP20 | RI | |  | | ZNF655 | | AT | | |
| THYN1 | RI |  | | USP35 | AP | |  | | ZNF675 | | AT | | |
| TIAM2 | AP |  | | USP45 | ES | |  | | ZNF684 | | AT | | |
| TIMM17B | ES |  | | USP6NL | AT | |  | | ZNF692 | | AA | | |
| TJP1 | ES |  | | USP8 | ES | |  | | ZNF7 | | ES | | |
| TLE1 | AT |  | | VAMP2 | RI | |  | | ZNF700 | | AP | | |
| TMBIM4 | AT |  | | VWA8 | AT | |  | | ZNF714 | | AA | | |
| TMCO6 | RI |  | | WASH4P | RI | |  | | ZNF721 | | AT | | |
| TMEM107 | RI |  | | WDR11 | RI | |  | | ZNF726 | | AT | | |
| TMEM116 | ES |  | | WDR25 | ES | |  | | ZNF738 | | AT | | |
| TMEM126B | ES |  | | WDR55 | RI | |  | | ZNF816 | | AT | | |
| TMEM18 | ES |  | | WDR6 | RI | |  | | ZNF846 | | ES | | |
| TMEM231 | AA |  | | WHSC1 | AT | |  | | ZNHIT3 | | ES | | |
| TMEM234 | AT |  | | WRAP73 | RI | |  | | ZSCAN25 | | AD | | |
| TMEM242 | AT |  | | WWP2 | AT | |  | | ZSWIM7 | | RI | | |
| TMEM25 | ES |  | | XAF1 | AA | |  | | ZWINT | | RI | | |
| TMEM44 | AT |  | | YIPF1 | ES | |  | | ZXDC | | AT | | |

Alternate acceptor (AA), alternate donor (AD), alternate promoter (AP), alternate terminator (AT), exon skip (ES), mutually exclusive exons (ME), retained intron (RI).
