# Supplementary material for: Radiation-induced alternative transcripts as detected in total and polysome-bound mRNA
Source: Oncotarget. 2017 Oct 9;9(1):691–705. doi: 10.18632/oncotarget.21672 (PMC5787501; doi:10.18632/oncotarget.21672)
Supplement: Supplementary file 4 [file oncotarget-09-691-s004.docx]

**Supplementary Table 3:**  **Alternative splice events in the transcriptome after radiation**

| **Gene Symbol** | **Splice Type** |  | **Gene Symbol** | **Splice Type** |
| --- | --- | --- | --- | --- |
| ARMCX3 | AA |  | PXK | AA |
| ATG16L2 | AD |  | QTRTD1 | ES |
| ATXN2 | ES |  | QTRTD1 | ES |
| BBS5 | ES |  | RCOR3 | AT |
| BICD1 | ES |  | RIF1 | RI |
| BRICD5 | RI |  | RNF146 | ES |
| BRPF1 | AD |  | SLCO4A1 | RI |
| BTN3A1 | AA |  | SMEK1 | AD |
| BUB3 | AA |  | SOS1 | ES |
| C11orf30 | ES |  | SREK1 | ES |
| C17orf53 | ES |  | SRRM1 | ES |
| C1orf63 | ES |  | STARD4 | ES |
| C8orf58 | ES |  | STRN3 | ES |
| CCNL1 | ES |  | SYNRG | ES |
| CPEB2 | ES |  | TANC2 | ES |
| DDX50 | ES |  | TGFBR2 | ES |
| DEDD2 | ES |  | THAP5 | AD |
| DTX2 | ES |  | TIAL1 | ES |
| E2F6 | ES |  | TLK2 | ES |
| FADS3 | RI |  | TMEM136 | AA |
| FAM13B | ES |  | TMEM161B | ES |
| FAM185A | AD |  | TNFRSF25 | AA |
| FBXL12 | ES |  | TNFRSF25 | AA |
| FBXL4 | ES |  | TNRC6A | ES |
| FOXN2 | ES |  | TOP3B | RI |
| FRMD6 | AD |  | TRIM33 | ES |
| FRS2 | ES |  | TRMT10B | AD |
| GLTSCR1 | ES |  | TRMT44 | AA |
| GMIP | ES |  | TRNT1 | AD |
| HEXIM2 | AP |  | TYW5 | AD |
| HIPK3 | ES |  | VPS9D1 | AP |
| KIF13B | ES |  | WDR20 | ES |
| LIN54 | AD |  | ZFAND1 | ES |
| MAGI1 | ES |  | ZIK1 | AD |
| MAP3K3 | ES |  | ZMYND8 | ES |
| METTL3 | RI |  | ZNF138 | ES |
| MYL5 | ES |  | ZNF140 | ES |
| NDEL1 | ES |  | ZNF227 | ES |
| NHSL1 | ES |  | ZNF232 | RI |
| NME6 | ES |  | ZNF234 | AD |
| NME6 | AA |  | ZNF418 | ES |
| OSBPL6 | ES |  | ZNF544 | AA |
| PAPD4 | AA |  | ZNF714 | AA |
| PAX6 | ES |  | ZNF816 | AD |
| PNISR | RI |  | ZNF85 | AT |
| PWWP2A | ES |  | ZSCAN30 | AD |

Including splice type. Splice events in red are common to both the transcriptome and the translatome.  Alternate acceptor (AA), alternate donor (AD), alternate promoter (AP), alternate terminator (AT), exon skip (ES), mutually exclusive exons (ME), retained intron (RI).
